# Magnitude of glycemic control and its associated factors among patients with type 2 diabetes at Tikur Anbessa Specialized Hospital, Addis Ababa, Ethiopia

**DOI:** 10.1371/journal.pone.0193442

**Published:** 2018-03-05

**Authors:** Yohannes Tekalegn, Adamu Addissie, Tedla Kebede, Wondimu Ayele

**Affiliations:** 1 Tikur Anbessa Specialized Hospital, College of Health Sciences, Addis Ababa University, Addis Ababa, Ethiopia; 2 School of Public Health, College of Health Sciences, Addis Ababa University, Addis Ababa, Ethiopia; 3 School of Medicine, College of Health Sciences, Addis Ababa University, Addis Ababa, Ethiopia; Universidad Miguel Hernandez de Elche, SPAIN

## Abstract

**Back ground:**

Diabetes is increasing at an alarming rate throughout the world and about 80% of diabetic cases live in low and middle income countries. Glycemic control is the most important predictor for diabetic related complications and deaths. Identifying factors associated with glycemic control help health care providers and patients to work in the areas that reduce risks of diabetic related complications and deaths.

**Objectives:**

The aim of this study is to assess the magnitude and factors associated with glycemic control among type 2 diabetic patients at Tikur Anbessa Specialized Hospital, Addis Ababa, Ethiopia.

**Methods:**

Hospital-based cross sectional study was conducted on 412 type 2 diabetic patients who were attending in diabetic clinics at Tikur Anbessa Specialized Hospital. Data were collected through structured interview questionnaire, and data abstraction format to collect information from each patient’s medical records from March to April, 2015. Data were entered and analyzed using SPSS version 20 statistical software. Both descriptive and inferential statistics were used to determine magnitude of glycemic control and factors associated with poor glycemic control.

**Result:**

Median age of participants was 52 years old (IQR = 40–60 years old). From the study participants,51.7% were females. Median duration of living with diabetes since diagnosis was 10 years (IQR: 5–16 years). About 80% of the respondents had uncontrolled fasting blood glucose level. The factors which are significantly associated with poor glycemic control were longer duration of diabetes (AOR = 2.72 95%CI:1.16–6.32), and being on insulin therapy (AOR = 3.01 95% CI: 1.5–5.9).

**Conclusion:**

A high proportion of patients had poor glycemic control. Longer duration of the disease, and being on drug regimen of insulin were associated with poor glycemic control. Appropriate attention should be given to patients with longer duration of disease and those who are on insulin therapy.

## Back ground

Diabetes (DM) is defined as a metabolic disorder of multiple etiologies characterized by chronic hyperglycemia with disturbance in carbohydrate, protein and fat metabolism resulting from defect in insulin secretion, insulin action or both [1,2,3,4]

In Ethiopia, International Diabetes Federation (IDF) reported about 1.9 million adults aged 20–79 years were estimated to have diabetes in 2013 and another 2.9 million people living with impaired glucose tolerance who are at higher risk of developing diabetes. With national diabetes prevalence of 4.36% and there was about 34,262 estimated diabetes related deaths occurred in same year[1].

Hyperglycemia and diabetes are important causes of mortality and morbidity worldwide, through both direct clinical consequences and increased mortality from cardiovascular and kidney diseases. Control of hyperglycemia is a major therapeutic objective for all diabetic patients in preventing complications arising from diabetes[5]. Several large prospective studies and clinical trials established the benefits of intensive diabetes management in reducing micro vascular complications of diabetes[6].

Despite the established facts that diabetes patients benefited from control of hyperglycemia [7,8,9,10], majority of the patients fail to achieve adequate level of glycemic control[11]. And reasons for poor glycemic control is complex and multi factorial[12]. Study conducted in Ethiopia among type 2 diabetes found that more than 70% of patients poorly controlled their diabetes HbA1c >8%[13]

According to a previous study conducted In Ethiopia only 5% of diabetic patients had access to self monitoring of blood glucose at home. And none of them had glycated hemoglobin (HbA1c) determinationand75% of the patients required admission directly or indirectly due to uncontrolled diabetes[14].

In recent years, non communicable diseases had been problems of developing country and contributing significant number of adult deaths in this region. Diabetes is one of the common non communicable disease with high prevalence and risks of lifelong chronic complications.

This study aims to assess the magnitude of glycemic control and factors contributed to poor glycemic control among type 2 diabetic patients.

## Materials and methods

### Study area and period

Hospital based cross sectional study was conducted among type 2 diabetes patients who were attending in Diabetic clinics at Tikur Anbessa Specialized Hospital (TASH), Ethiopia. The hospital is found in Lideta Sub City, Addis Ababa, Ethiopia. It is the largest tertiary hospital with specialized service for diabetic patients. Patients were referred to this hospital from other health institution across the country. The endocrinology unit in the hospital had two clinics visit schedule every week for patients with type 2 diabetes and average numbers of patients attending the clinic in one month were estimated to a number of 526. This study was conducted from March to April, 2015 at diabetic clinic of the hospital.

### Participant’s eligibility criteria

Diabetic Patients aged greater than or equals to 15 years old with regular follow up, and had at least 3 or more measurements of fasting blood sugar (FBS) level in past year, were included in the study. Participants who are not willing to participate in the study are excluded from the study.

### Sample size determination

The required sample size for the study is estimated using the proportion of diabetics with poor glycemic control, 50% which was reported from study conducted in Ambo hospital among type 2 diabetic patients[15].95% level of confidence (α) and 80% power (ß) is used. Including 10% non response rate, the calculated sample size was 422.

$$\text{n}=\frac{{\text{(}z_{\alpha /2})}_{}^{2}\times pq}{d_{}^{2}}$$

Where:

n = the desirable sample size

Z (α/2) = the critical value at 95% level of significance (1.96)

p = proportion of patients with poor glycemic control

d = precision of measurement (acceptable marginal error)

p = 0.5

d = 0.05

$$\text{n}=\frac{{\left(1.96\right)}_{}^{2}\times 0.5*0.5}{{\left(0.05\right)}_{}^{2}}=384$$

Estimating10%nonresponserate=(0.1)*(384)=38.4

384+38.4=422

### Data collection procedure

Purposively, Tikur Anbessa Specialized Hospital (TASH) was selected as study area. Systematic random sampling technique was used to select the study subjects. Eligible study participants were interviewed face to face using structured data collection tools. In addition data abstraction format was used to collect information from participants’ medical records. The tools contain information about socio-demographic characteristics of the patients, self care activities, clinical, behavioral, psychological characteristics and checklist to review patients’ medical record. The data collection tools were first prepared in English and then translated to Amharic. Finally, translated back to English by different expert to ensure validity of translation.

### Data collection tools

**Part 1**: Questionnaires on socio demographic variables were prepared, and study participants were interviewed face to face.

**Part 2**: Tools to asses self care activities of the patients: Summary of diabetes self care activities (**SDSCA**) scale is used. This scale was developed by Toobert and Glasgow; it has acceptable reliability and validity. It contains 12 questions about the diet, exercises, blood sugar test, foot care, and medication. Patients were interviewed face to face for each question[16].

**Part3**: Tools to assess diabetic distress level of the study participants: The diabetic distress score (DDS) was used. This scale is developed by Fisher and his colleague[17]. Diabetic distress score (**DDS17**) which is composed of 17 questions was used to explore contents of diabetic distress among study participants.

**Part 4**: Checklist to review patient’s medical record: After the patients had completed their interviews their respective medical record were reviewed using a check list to obtain their last three fasting blood glucose, type of treatment regimen patient were receiving.

### Data management and quality assurance

To ensure data quality, reliability of data extraction forms were checked by doing pre test on 5% of the sample size, training was given for data collectors. Accuracy and completeness of data were checked daily after data collection time. For data entry and analysis SPSS version 20 was used. The study participants were dichotomized based on their fasting blood glucose (FBG) in to controlled glycaemia (FBG 70-130mg/dl) and uncontrolled glycaemia (FBG<70 mg/dl and >130 mg/dl). Adherence to diabetes self care was categorized into two categories (adherent and non-adherent) based on their average score, diabetes distress into (moderate distress and no distress) categories based on average scores. After categorization was completed, each variable is checked for missed values, and normality test is performed.

### Data analysis procedures

Descriptive statistics like frequency, proportion, mean, median and standard deviation were employed in describing socio demographic, clinical, and behavioral characteristics of patients. Odds ratio was used to assess association between poor glycemic control and independent categorical variables. Variables found significant at p-value <0.05 in bivariate analysis were included in to multivariate logistic regression analysis. Statistical significance was set at p< 0.05.

### Ethical statement

Prior to data collection ethical clearance was obtained from institutional review board (IRB) of Addis Ababa University, College of Health Sciences, School of Public Health. Information sheet explaining the aim of the study prepared and read to all eligible participants. Informed consent was obtained from all study participants. For participants aged below 18 years old, Informed consent was received from the parent or legal guardian of the participants.

## Operational definition

**Glycemic control**: For the purpose of this study, we categorized the study participants in to two groups based on the American Diabetes Association(ADA) recommendation [18]:

- Good glycemic control: fasting blood glucose of 70–130 mg/dl[18].
- Poor glycemic control: fasting blood glucose of <70mg/dl and >130mg/dl[18].

**Fasting blood sugar**: Blood glucose measured from venous blood after 8hours of overnight fasting or longer.

**Diabetes distress**: If the study participant’s mean item score for “DDS17” is ≥3, considered as a level of distress worthy of clinical attention.

**Adherence to medication**: if the study participant took all his/her anti diabetic medication in the last seven days.

**Adherence to diet**: If the study participant had followed the recommended diet for 3 or more days in last seven days.

**Adherence to exercise**: If the study participant had followed the recommended level of exercise for 3 or more days in last seven days.

**Regular follow up**: a type 2 diabetic patient registered at endocrinology unit of the hospital for follow up

## Result

### Socio-demographic characteristics

Of the total of 422 sample size 412 respondents’ case is included in the data analysis with response rate of 97.6%.10 cases with incomplete documents were excluded from the data analysis. Out of 412 respondents, 213(51.7%) and 189(49.3%) were females and males respectively. The median age of respondents is 52 years old (IQR 40–60 years old). Out of the total, 281(68.2%) respondents were married. Majority 325(78.9%) of the respondents were Orthodox Christian. 77(18.7%) were found to be illiterate, and 125(30.3%) had completed grade 12^th^ and above. In terms of employment120 (29.1%) were un- employed, 120(29.1%) were government or private employees, 121(29.4%) self employed and 50(12.1%) were retired (Table 1).

**Table 1:** Socio-demographic characteristics of study participants(type 2 diabetes patients, n = 412); at Tikur Anbessa Specialized Hospital (TASH), Addis Ababa, Ethiopia, 2015.

Characteristics / variables	Frequency	Percentage
**Sex**		
Male	199	48.3
Female	213	51.7
**Age**		
<40	96	23.3
40–49	73	17.7
50–59	123	29.9
≥60	120	29.1
**Educational status**		
Illiterate	77	18.7
1–6	114	27.7
7–12	96	23.3
>12	125	30.3
**Marital status**		
Single	74	18
Married	281	68.2
widowed	37	9
Divorced/separated	20	4.8
**Religion**		
Orthodox	325	78.9
Muslim	35	8.5
Protestant	42	10.2
Others	10	2.4
**Ethnicity**		
Amhara	229	55.6
Oromo	37	9.1
Gurage	89	22
Tigre	25	6.2
Others	25	6.2
**Occupation**		
Unemployed	121	29.4
Government or private employee	120	29.1
Self employed	121	29.4
Retired	50	12.1
**Income**		
≤1000 birr	123	52.1
>1000 birr	112	47.9

### Self-care behaviors of the study participants

Of the total 412 respondents, 224(54.4%) were taking adequate physical exercise and 350(85%) were not testing their blood glucose level adequately.257 (57.5%) of the respondents were not following their general dietary program correctly. 357 (86.7%) respondents were taking their medication as recommended by their doctors(Table 2).

**Table 2:** Summary of diabetic self care activities (SDSCA) of the study participants, TASH, Addis Ababa, Ethiopia,2015.

Variables	number	Percent
**Compliance to general diet program the in last seven days**		
>3 days (adequate)	175	42.5
0–3 days (in adequate)	237	57.5
**Compliance to specific diet program in the last seven days**		
>3 days (adequate)	312	75.7
0–3 days (in adequate)	100	24.3
**Physical exercise in the last seven days**		
>3 days (adequate)	224	54.4
0–3 days (in adequate)	118	45.6
**Compliance to blood sugar testing in the last seven days**		
>3 (adequate)	62	15
0–3 (in adequate)	350	85
**Compliance medication in the last seven days**		
7 days (adequate)	357	86.7
< 7 days (in adequate)	55	13.3

### Knowledge, behavioral and clinical characteristics of the respondents

307 (74.5%) of the respondents did not know their target blood glucose level for diabetes management. 335 (81.3%) of the respondents had no adequate knowledge about signs and symptoms of hyper and hypoglycemia.

196(47.6%) of the respondents had never attended any diabetic education session. Of the total respondents, 298(72.3%) had less than three times follow up in the clinic per year.

Concerning alcohol consumption, 90(20.2%) had a history of intake before 12 months from the time of the survey. Regarding smoking, 8(2%) had a history of smoking in the last seven days prior to the date of survey.

Median duration of diabetes is 10 (IQR 5–16) years. A total of 303(97.8%) respondents were on pharmacological therapy for diabetes.

Out of those who were on medication for diabetes, 237(57.5%) respondents were taking insulin alone; 128(31.1%) were taking oral hypoglycemic agents and the rest were on both insulin and oral hypoglycemic agents (Table 3).

**Table 3:** Knowledge, behavioral and clinical characteristics of the respondents at TASH, Addis Ababa, Ethiopia, 2015.

Variables	frequency	Percentage
**Ever attended diabetic education**		
Yes	216	52.4
No	196	47.6
**Number of follow up to diabetic clinic per year**		
≤3	298	72.3
>3	114	27.7
**Number diabetic education Sessions ever attended n = 215**		
1–2 times	124	57.6
≥3 times	91	42.4
**Knowledge of target blood glucose level**		
Yes	105	25.5
No	307	74.5
**Knowledge of sign and symptoms of hyper and hypoglycaemia**		
Yes	77	18.7
No	335	81.3
**Alcohol consumption**		
Yes	90	21.8
No	322	78.2
**Smoking**		
Yes	8	2
No	404	98
**Duration of diabetes**		
<5 years	91	22.1
5–10 years	121	29.4
>10 years	200	48.5
**Drug regimen**		
Oral hypoglycaemic agent(OHA)	128	31.1
Insulin	237	57.5
Insulin and oral hypoglycaemic agents	38	9.2

### Diabetic distress

Of total 412 interviewed study participants, one hundred sixty (38.8%) had moderate diabetic distress worthy of clinical attention. Mean diabetic distress score is 2.68±1.09 SD. Each sub scale of diabetic distress is described below (Table 4).

**Table 4:** Diabetic distress and its subscale among type 2 diabetes patients at TASH, Addis Ababa, Ethiopia, 2015.

Variables	Frequency	Percent
**Diabetic distress**		
No	252	61.2
Yes	160	38.8
**Emotional distress**		
No	189	45.9
Yes	223	54.1
**Physician related distress**		
No	284	68.9
Yes	128	31.1
**Regimen related distress**		
No	242	58.7
Yes	170	43.3
**Interpersonal distress**		
No	275	66.7
Yes	137	33.3

### Magnitude of glycemic control

**Fig 1:**
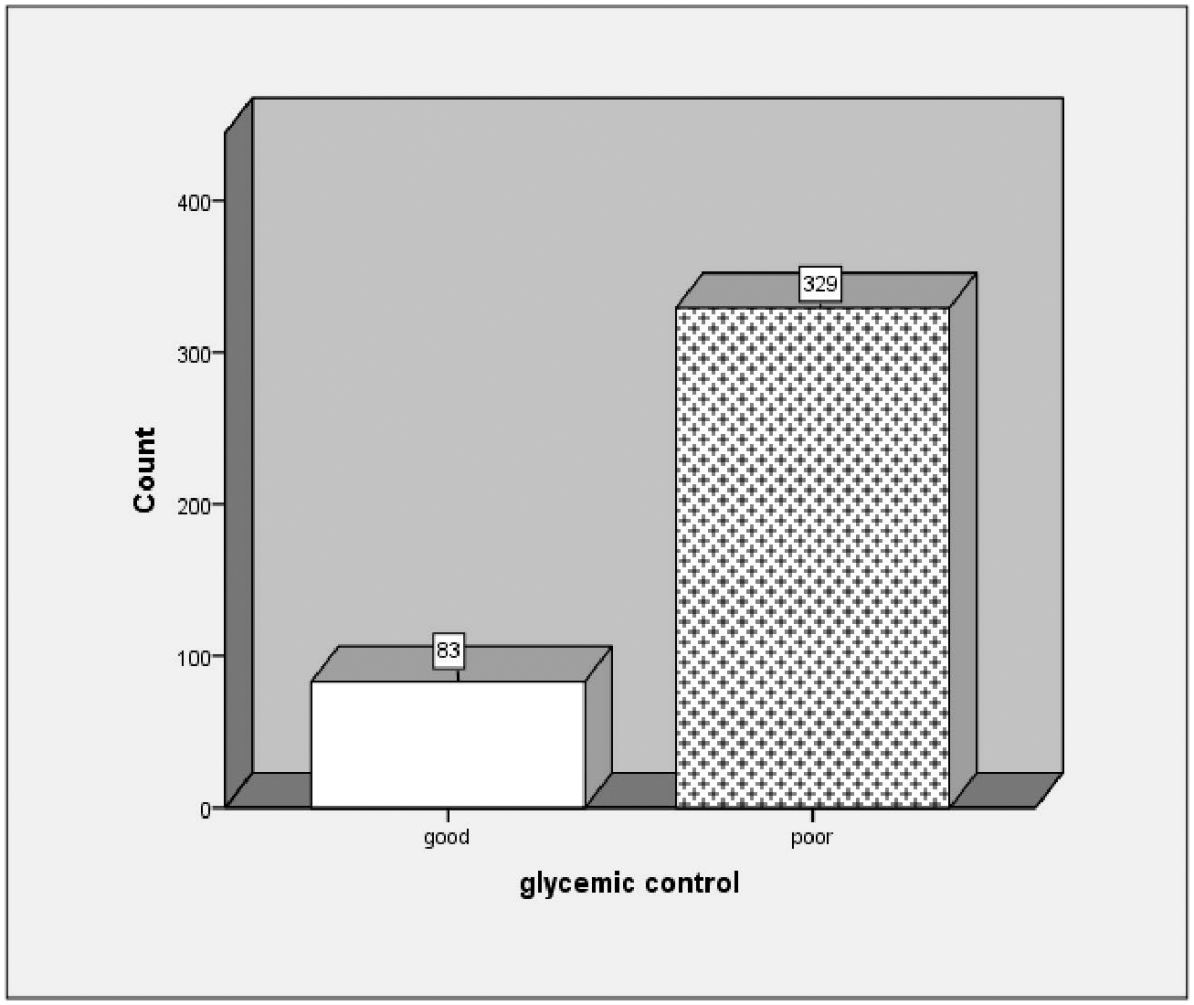
Magnitude of glycemic control among type 2 diabetes patients at TASH, Addis Ababa, Ethiopia, 2015.

Mean fasting blood glucose from the last three clinic visits were used to determine glycemic control. Mean fasting blood glucose(FBS) of the respondents was 165.63 mg/dl ±51.82 mg/dl. Proportion of patients with poor glycemic control at level of FBS >130 mg/dl and FBS <70 mg/dl was 83(80%) [95% CI: 75.7%-83.5%], most 324(78.6%) had FBS >130 mg/dl, and only five (1.2%) had FBS<70 mg/ dl (Fig 1).

### Factors associated with glycemic control

Bivariate logistic regression revealed that age, duration of diabetes, and drug regimens were associated with glycemic control (Table 4).

After controlling of potential confounding factors, drug regimen and duration of disease were associated with glycemic control.

Odds of poor glycemic control of diabetic patients who were on insulin therapy were three times higher (AOR = 3.0, 95% CI: 1.5, 5.9) comparedto patients who were using drug regimen oral hypoglycemic agent alone.

Being a diabetic patient with a duration of 5–10 years has 2.72 times higher (AOR = 2.72, 95% CI: 1.16, 6.32) risk for poor glycemic control compared to a patient who was living with diabetes with a duration of less than 5 years (Table 5).

**Table 5:** Multivariate logistic regression of factors associated with glycemic control among type 2 DM patients at TASH, Addis Ababa, Ethiopia, 2015.

Variables	Number (%)	COR (95%CI)	AOR (95% CI)
**Age**			
<40	96(23.3)	1	1
40–49	73(17.7)	0.824(0.36,1.88)	2.14(0.74,6.2)
50–59	123(29.9)	0.797(0.38,1.64)	2.46(0.91,6.63)
≥60	120(29.1)	0.48(0.25,0.92)	1.02(0.37,2.78)
**Duration of diabetes**			
<5 years	91(22.1)	1	1
5–10 years	121(29.4)	2.98(1.42,6.02)	2.72(1.16,6.32)*
>10 years	200(48.5)	1.63(0.92,2.88)	1.7(0.8,3.7)
**Drug regimen**			
Oral hypoglycaemic agents (OHA)	128(31.1)	1	1
Insulin	237(57.5)	2.35(1.4,3.96)	3.01(1.5,5.99)*
Insulin and OHA	38(9.2)	0.44(0.17,1.14)	1.2(0.24,6.27)
Diet only	9(2.2)	1.18(0.28,4.98)	2.9(0.86,9.9)

* Significant at p<0.05

**COR**: crude odds ratio; **AOR**: adjusted odds ratio

## Discussion

This study assessed the magnitude of glycemic control and its associated factors among type 2 diabetes patients at Tikur Anbessa Specialized Hospital (TASH), Addis Ababa, Ethiopia. The study found that, over all, glycemic control among the study subjects was far below the recommended standards. The mean fasting blood glucose level of the study subjects were 165.63±51.82 mg/dl. This finding is comparable with a study in Jimma, Ethiopia where mean fasting blood glucose was 171±63 mg/dl [19], And also in line with a study in Addis Ababa where mean fasting blood glucose was 190±89.6 mg/dl[20]. Evidently, our finding is much higher than the American Diabetic Association recommendation[18].

None of the patients had HbA1c determination: similar with previous studies from Jimma, Ethiopia [19,21] and Addis Ababa, Ethiopia[20], due to unavailability of the laboratory service for the HbA1c determination in the public health institutions of Ethiopia.

This study found that a high proportion (80%) of study participants had poor glycemic control. This study finding is comparable to a study conducted in Jimma, where proportion of patients with poor glycemic control was82% and81.7% [19,22].Proportion of poor glycemic control in the present study is much higher than the studies conducted in Amman Jordan, Ambo and Gondar where poor glycemic control is 65.1%,64.7%, and 50%[15,23,24]. The possible explanation for this difference could be that the patients seeking advanced management were referred to Tikur Anbessa Specialized Hospital. It is the only hospital in the country where patients were referred to but coming from the whole regions of the country.

This study found that only 19.7% of the patients had adequate knowledge about signs and symptoms of hyper and hypoglycemia. This finding is lower than the finding from the study conducted in Jimma, Ethiopia where about 70% of the patients had adequate knowledge about sign and symptoms of hyperglycemia[22]. This variation could be related to a difference in the scoring and categorization of knowledge question items; where this study used a mean score of knowledge item questions to categorize respondents in to adequate and inadequate knowledge level whereas the former study used 60% score and above as satisfactory knowledge level.

Practice of self monitoring for blood glucose level at home is low in the present study. This finding is similar with previous studies, 5.5% in Addis Ababa and 5% in Jimma[19,20]. This could be related with financial capacity of the patients to buy glucometer and strips at the study areas.

Knowledge towards target blood glucose level for diabetes management found to be very low 25.5%. This finding is similar with a study from jimma[21].It indicates that patients solely depend on their health care providers’ assistance to control their diabetes. It is difficult for patients to take appropriate measures without knowing the target of the treatment. Unless patients understand the chronic nature of the disease and actively gets involved in their treatment process it would be difficult to attain adequate level of normal glycemia.

The study found that patients who were on insulin were more likely to poorly control their fasting blood glucose level (AOR = 3.01 95% CI: 1.5–5.99) when compared to patients taking oral hypoglycemic agents alone. This finding is consistent with other similar studies [21,25], Usually type 2 DM patients with good glycemic control stay on oral hypoglycemic agents and those with poor glycemic control most likely upscale to insulin therapy. This could explain why the number of patients with poor glycemic control was higher among insulin user patients. Adherence to insulin self injection might also be less than the adherence to oral hypoglycemic agents due to different factors.

The study found that patients Who Contracted a longer duration (5–10 years) of diabetes were more likely (AOR = 2.72 95% CI: 1.16–6.32) to have poor glycemic control compared with patients with relatively shorter (< 5 years) duration of diabetes. Also this may be explained by adherence of patients with shorter duration of the disease to medication and diet. This finding is consistent with several previous studies[26,27].

## Conclusion

This study revealed that 80% of patients poorly controlled their diabetes. Knowledge towards sign and symptom of hyper and hypoglycemia and knowledge about target blood glucose is meager. On the other hand this affects the patients’ active involvement in appropriate diabetes self-care. This study also found that majority of patients did not attend health education provided at the hospital. Percentage of patients practicing self monitoring of blood glucose was very low; this could lead to delay in making appropriate corrective actions at their home during their routine daily activities before going place where health service is rendered.

After controlling the confounding factors, longer duration of diabetes and engaging on insulin therapy were associated with poor glycemic control. Special attention should be given for patient with a longer duration of diabetes and those who are taking insulin therapy.

## Supporting information

S1 Table Socio-demographic characteristics of study participants(type 2 diabetes patients, n = 412); at Tikur Anbessa Specialized Hospital (TASH), Addis Ababa, Ethiopia, 2015. (DOCX)

S2 Table Summary of diabetic self care activities (SDSCA) of the study participants, TASH, Addis Ababa, Ethiopia,2015. (DOCX)

S3 Table Knowledge, behavioral and clinical characteristics of the respondents at TASH, Addis Ababa, Ethiopia, 2015. (DOCX)

S4 Table Diabetic distress and its subscale among type 2 diabetes patients at TASH, Addis Ababa, Ethiopia, 2015. (DOCX)

S5 Table Multivariate logistic regression of factors associated with glycemic control among type 2 DM patients at TASH, Addis Ababa, Ethiopia, 2015. (DOCX)
