# Supplementary material for: Magnitude of glycemic control and its associated factors among patients with type 2 diabetes at Tikur Anbessa Specialized Hospital, Addis Ababa, Ethiopia
Source: PLoS One. 2018 Mar 5;13(3):e0193442. doi: 10.1371/journal.pone.0193442 (PMC5837131; doi:10.1371/journal.pone.0193442)
Supplement: S1 Table — (DOCX) [file pone.0193442.s001.docx]

| **Characteristics / variables** | **Frequency** | **Percentage** |
| --- | --- | --- |
| **Sex** |  |  |
| Male | 199 | 48.3 |
| Female | 213 | 51.7 |
| **Age** |  |  |
| <40 | 96 | 23.3 |
| 40-49 | 73 | 17.7 |
| 50-59 | 123 | 29.9 |
| ≥60 | 120 | 29.1 |
| **Educational status** |  |  |
| Illiterate | 77 | 18.7 |
| 1-6 | 114 | 27.7 |
| 7-12 | 96 | 23.3 |
| >12 | 125 | 30.3 |
| **Marital status** |  |  |
| Single | 74 | 18 |
| Married | 281 | 68.2 |
| widowed | 37 | 9 |
| Divorced/separated | 20 | 4.8 |
| **Religion** |  |  |
| Orthodox | 325 | 78.9 |
| Muslim | 35 | 8.5 |
| Protestant | 42 | 10.2 |
| Others | 10 | 2.4 |
| **Ethnicity** |  |  |
| Amhara | 229 | 55.6 |
| Oromo | 37 | 9.1 |
| Gurage | 89 | 22 |
| Tigre | 25 | 6.2 |
| Others | 25 | 6.2 |
| **Occupation** |  |  |
| Unemployed | 121 | 29.4 |
| Government or private employee | 120 | 29.1 |
| Self employed | 121 | 29.4 |
| Retired | 50 | 12.1 |
| **Income** |  |  |
| ≤1000 birr | 123 | 52.1 |
| >1000 birr | 112 | 47.9 |
