# Supplementary material for: Magnitude of glycemic control and its associated factors among patients with type 2 diabetes at Tikur Anbessa Specialized Hospital, Addis Ababa, Ethiopia
Source: PLoS One. 2018 Mar 5;13(3):e0193442. doi: 10.1371/journal.pone.0193442 (PMC5837131; doi:10.1371/journal.pone.0193442)
Supplement: S2 Table — (DOCX) [file pone.0193442.s002.docx]

| **Variables** | **number** | **Percent** |
| --- | --- | --- |
| **Compliance to general diet program the in last seven days** |  |  |
| >3 days ( adequate) | 175 | 42.5 |
| 0-3 days (in adequate) | 237 | 57.5 |
| **Compliance to specific diet program in the last seven days** |  |  |
| >3 days ( adequate) | 312 | 75.7 |
| 0-3 days (in adequate) | 100 | 24.3 |
| **Physical exercise in the last seven days** |  |  |
| >3 days ( adequate) | 224 | 54.4 |
| 0-3 days (in adequate) | 118 | 45.6 |
| **Compliance to blood sugar testing in the last seven days** |  |  |
| >3 ( adequate) | 62 | 15 |
| 0-3 (in adequate) | 350 | 85 |
| **Compliance medication in the last seven days** |  |  |
| 7 days (adequate) | 357 | 86.7 |
| < 7 days ( in adequate) | 55 | 13.3 |
