# Supplementary material for: Magnitude of glycemic control and its associated factors among patients with type 2 diabetes at Tikur Anbessa Specialized Hospital, Addis Ababa, Ethiopia
Source: PLoS One. 2018 Mar 5;13(3):e0193442. doi: 10.1371/journal.pone.0193442 (PMC5837131; doi:10.1371/journal.pone.0193442)
Supplement: S3 Table — (DOCX) [file pone.0193442.s003.docx]

| **Variables** | **frequency** | **Percentage** |
| --- | --- | --- |
| **Ever attended diabetic education** |  |  |
| Yes | 216 | 52.4 |
| No | 196 | 47.6 |
| **Number of follow up to diabetic clinic per year** |  |  |
| ≤3 | 298 | 72.3 |
| >3 | 114 | 27.7 |
| **Number diabetic education Sessions ever attended n =215** |  |  |
| 1-2 times | 124 | 57.6 |
| ≥3 times | 91 | 42.4 |
| **Knowledge of target blood glucose level** |  |  |
| Yes | 105 | 25.5 |
| No | 307 | 74.5 |
| **Knowledge of sign and symptoms of hyper and hypoglycaemia** |  |  |
| Yes | 77 | 18.7 |
| No | 335 | 81.3 |
| **Alcohol consumption** |  |  |
| Yes | 90 | 21.8 |
| No | 322 | 78.2 |
| **Smoking** |  |  |
| Yes | 8 | 2 |
| No | 404 | 98 |
| **Duration of diabetes** |  |  |
| <5 years | 91 | 22.1 |
| 5-10 years | 121 | 29.4 |
| >10 years | 200 | 48.5 |
| **Drug regimen** |  |  |
| Oral hypoglycaemic agent(OHA) | 128 | 31.1 |
| Insulin | 237 | 57.5 |
| Insulin and oral hypoglycaemic agents | 38 | 9.2 |
