# Supplementary material for: Magnitude of glycemic control and its associated factors among patients with type 2 diabetes at Tikur Anbessa Specialized Hospital, Addis Ababa, Ethiopia
Source: PLoS One. 2018 Mar 5;13(3):e0193442. doi: 10.1371/journal.pone.0193442 (PMC5837131; doi:10.1371/journal.pone.0193442)
Supplement: S4 Table — (DOCX) [file pone.0193442.s004.docx]

| **Variables** | **Frequency** | **Percent** |
| --- | --- | --- |
| **Diabetic distress** |  |  |
| No | 252 | 61.2 |
| Yes | 160 | 38.8 |
| **Emotional distress** |  |  |
| No | 189 | 45.9 |
| Yes | 223 | 54.1 |
| **Physician related distress** |  |  |
| No | 284 | 68.9 |
| Yes | 128 | 31.1 |
| **Regimen related distress** |  |  |
| No | 242 | 58.7 |
| Yes | 170 | 43.3 |
| **Interpersonal distress** |  |  |
| No | 275 | 66.7 |
| Yes | 137 | 33.3 |
