# Supplementary material for: Magnitude of glycemic control and its associated factors among patients with type 2 diabetes at Tikur Anbessa Specialized Hospital, Addis Ababa, Ethiopia
Source: PLoS One. 2018 Mar 5;13(3):e0193442. doi: 10.1371/journal.pone.0193442 (PMC5837131; doi:10.1371/journal.pone.0193442)
Supplement: S5 Table — (DOCX) [file pone.0193442.s005.docx]

| **Variables** | **Number (%)** | **COR( 95%CI)** | **AOR (95% CI)** |
| --- | --- | --- | --- |
| **Age** |  |  |  |
| <40 | 96(23.3) | 1 | 1 |
| 40-49 | 73(17.7) | 0.824(0.36,1.88) | 2.14(0.74,6.2) |
| 50-59 | 123(29.9) | 0.797(0.38,1.64) | 2.46(0.91,6.63) |
| ≥60 | 120(29.1) | 0.48(0.25,0.92) | 1.02(0.37,2.78) |
| **Duration of diabetes** |  |  |  |
| <5 years | 91(22.1) | 1 | 1 |
| 5-10 years | 121(29.4) | 2.98(1.42,6.02) | **2.72(1.16,6.32) ⃰** |
| >10 years | 200(48.5) | 1.63(0.92,2.88) | 1.7(0.8,3.7) |
| **Drug regimen** |  |  |  |
| Oral hypoglycaemic agents (OHA) | 128(31.1) | 1 | 1 |
| Insulin | 237(57.5) | 2.35(1.4,3.96) | **3.01(1.5,5.99) ⃰** |
| Insulin and OHA | 38(9.2) | 0.44(0.17,1.14) | 1.2(0.24,6.27) |
| Diet only | 9(2.2) | 1.18(0.28,4.98) | 2.9(0.86,9.9) |
